# Political correctness and the alt-right: The development of extreme political attitudes

**DOI:** 10.1371/journal.pone.0239259

**Published:** 2020-10-07

**Authors:** Jordan T. Moss, Peter J. O’Connor

**Affiliations:** 1 School of Psychology and Counselling, Queensland University of Technology, Brisbane, Australia; 2 School of Management, QUT Business School, Queensland University of Technology, Brisbane, Australia; Rice University, UNITED STATES

## Abstract

Recent studies have documented a shift from moderate political attitudes to more extreme attitudes at the ends of the political spectrum. This can be seen in Political Correctness (PC) on the left, and white identitarian (WI) attitudes on the ‘Alt-Right’ (AR). While highly covered in mainstream media, limited academic research has investigated their possible antecedents and psychological correlates. The current study investigated the prevalence and psychological predictors of these attitudes. Utilising a quota-based sample of 512 U.S. participants, we found that extreme political attitudes were associated with various personality traits, social media use, and upbringing. PC attitudes were associated with agreeableness, black-white thinking, social-media use, and perceived overprotective parenting. WI attitudes were associated with low agreeableness and openness, and high black-white thinking. Our results show that extreme left and right attitudes are separated by individual differences, and that authoritarianism can be seen on both the left and the right.

## Introduction

Political attitudes are typically measured on a single continuum with ‘left-wing’ ideologies advocating for liberal values, and ‘right-wing’ ideologies promoting conservative principles. Traditionally, U.S. political opinion has been largely situated in the centre, with the electorate holding a mix of liberal and conservative attitudes [1]. However, recent political commentary has suggested an evacuation from moderate views and a move toward more extreme attitudes [2]. This migration has manifested on both sides of the political aisle, as left and right have fragmented with the inscription of Political Correctness (PC) in the so-called ‘Regressive Left’, and white identitarian (WI) attitudes in the ‘Alt-Right’ (AR) [3, 4].

Mainstream news outlets have emphasized the role that adherents to such ideologies have had in violent incidences such as the Charlottesville rally [5] and numerous protests on college campuses over free speech [6, 7]. Authors have argued that these movements are associated with deleterious societal outcomes, including discrimination [5] and loss of free speech [8]. Accordingly, a 2018 survey found that 80% of Americans consider PC an issue and 60% consider white supremacists a growing threat in the U.S. [9]. While highly covered in the media, little research has investigated the predictors of such attitudes.

Despite this, scholars and political commentators have theorized that the increase in PC is attributable to social media usage and changes in child rearing, with increased overprotective parenting [10]. Although widely publicized, limited peer-reviewed research has directly investigated these claims. Only two studies have investigated predictors of PC. Firstly, Andary-Brophy found that PC is partially explained by personality traits, specifically, high trait Agreeableness [11]. Further, PC was found to be a multidimensional construct, consisting of both liberal and authoritarian attitudes. Secondly, Moss & O’Connor reported that the Dark Triad traits and Entitlement explain a substantial amount of variance in the authoritarian attitudes of PC and WI [12].

While some authors have extended the effect of social media to explain the increase in extreme right attitudes [13, 14], there is no published investigation into such correlates of WI attitudes. The current study assesses whether a set of hypothesized factors predict these extreme political attitudes. By surveying a nationally quota-based sample of 512 U.S. participants on a range of personality, social, and political factors, we assess elements of Lukianoff and Haidt’s theory regarding PC attitudes and extend the study of political attitudes to those of the extreme right [10]. Specifically, we investigate 1) the extent to which PC and WI attitudes are held within the general U.S. population, 2) the possible antecedents of these attitudes, 3) whether ‘extremists’ on the left and right have traits in common, and 4) the claim that PC is a multidimensional construct. We argue that understanding the antecedents of extreme political attitudes will help clarify the etiology of ideological commitments in general and assist efforts to reduce the negative behaviours associated with these attitudes.

### Political attitudes and ideologies

Political attitudes have typically been conceptualised on a single left-right (liberal-conservative) continuum. Because political views are multidimensional, several authors have considered this one-dimensional measure inadequate [15, 16]; however, it demonstrates satisfactory predictive validity on voting behaviour [17]. The scale essentially measures one’s tendency toward advocating or resisting social change, and accepting or rejecting inequality [18].

Left voters are more likely to reject inequality and seek out change [18]. Historically, liberals have been considered as having an optimistic view of human nature [19]. Sowell has argued that liberals assume that perfectibility in man is achievable, which motivates an expanse to promote the ideal [20]. Contrasting this on the right, conservatives have been considered as having a more pessimistic view of human nature [19]. Contending that civility necessitates the constraints of authority and institutions, conservatism is concerned with the preservation of tradition and stability. Conservatives are more sensitive to threats against social order, which motivates a defence of social organizations and an insistence on hierarchy [21]. As such, conservatives are more likely to accept inequality and resist social change [18].

Although on different sides of the political spectrum, reports had previously situated both parties close to the centre [2]. However, with the aforementioned evacuation from moderate attitudes, the current political spectrum shows a less populated centre (which is becoming more vacuous) defining a chasm between the left and right. In a nationally representative study of 10,013 participants, the Pew Research Center found that, over the past two decades, fewer U.S. citizens are holding a mix of liberal and conservative attitudes [2]. In this study, participants answered questions about their political attitudes and engagement via a telephone interview or online survey. The authors found that twice as many Americans ubiquitously express opinions consistent with their party’s platform as they did in the 1990’s (from 10% in 1994 to 21% in 2014). Ideological uniformity was most pronounced for those most politically active, however the general overlap between the major parties has diminished. Beyond disconnecting from the other party, growing ideological uniformity also increased feelings of antipathy for the opposing party [1].

### Political correctness

The term PC is generally interpreted as any effort to avoid or suppress potentially offensive content [22]. While historically defined as the unequivocal commitment to party propaganda [23], reinterpreted on the principles of egalitarianism, PC is now solely (colloquially) associated with the left [11]. Proponents of PC (sometimes referred to as ‘social justice warriors’) have popularized terms such as ‘*trigger warnings’* (alerts that warn an audience of forthcoming content that may ‘trigger’ a remembrance of past trauma) and ‘*microaggressions*’ (small actions or statements that, though seemingly innocuous, are insensitive to another person’s felt experience) that encourage people to reassess their unassuming word choices, and accommodate for the emotional experience of others [10].

While a significant proportion of PC advocates argue that their motivation is compassion for others, scholars have noticed that methods used by some advocates involve purposeful violence and intimidation [24]. In the first empirical investigation of PC, Andary-Brophy found that PC is a two-factor structure, consisting of liberal and authoritarian components [11]. PC-Liberalism (PCL) represents the concern of individual welfare. PCL proponents are primarily concerned with promoting socially disadvantaged groups. They take offense to language that undermines their goal of diversity, and protest against the use of such language. PC-Authoritarianism (PCA) focuses on purity and safety. While promoting diversity, adherents of PCA appear to be primarily concerned with censoring language that is emotionally upsetting. Like PCL, PCA proponents protest against the use of offensive or exclusive language, however they show a greater inclination toward immediate, autocratic methods, typical of the aforementioned violent protests.

Although the concept of PC has not been well-substantiated in peer-review, Brophy’s (2015) conceptualization allows one to distinguish between the socially liberal effort towards cultural inclusivity and sensitivity (PCL), with the ‘cancel culture’ (PCA) that makes news headlines and represents the colloquial view of PC. These scales were found to be internally reliable (both reporting α > .8) which encourages the first steps toward measuring this previously abstract topic. Essentially, PC represents a sensitivity to offensive content, PCL represents sensitivity in content that challenges social inclusivity, whereas PCA represents sensitivity to content that challenges one’s own feelings of emotional safety.

### The ‘alt-right’ and white identitarian attitudes

The AR is a term used by the media and political commentators to describe several subgroups on the extreme right that reject mainstream conservative views [4]. The term has been given traction by highly influential people, such as the previous senior counselor to President Donald Trump, Steve Bannon. Considering traditional conservatism politically inefficient and culturally out-of-touch, advocates promote the AR as an alternative to conservative ideology [14]. While this vague definition can encompass any conservative disenchanted with their political representation, political commentators and figureheads of the ideology have specified that the AR essentially represents WI attitudes [3, 25].

In the promotion of WI politics, adherents of the AR indicate: 1) strong feelings of white identity, 2) a strong sense of white solidarity, and 3) a belief in white victimization [3]. Such attitudes have long been recognised to manifest in voting behavior, and political orientation. Kinder & Sears investigated the voting behavior of suburban white voters in two LA mayoral elections in 1969 and 1973, and how direct or socioculturally prejudiced racial threats motivated political decisions [26]. Direct racial threats were defined as how white participants considered the impact that blacks have on their personal life (schooling, jobs, neighborhoods). Sociocultural prejudice was considered more abstractly as ‘symbolic racism’: moralistic resentment, and alienation of blacks. While direct threats were minimally influential, symbolic racism was found to significantly skew white voters against black candidates. Furthermore, the authors found that one’s proximity and engagement with black neighbors had a non-significant effect in participants holding symbolically racist attitudes. This suggests that WI ideology represents deeper, moral claims, rather than private/ personal interactions.

Indeed, in several interviews and public statements, AR leaders announced Western culture as an achievement of white ingenuity, and that a defense of Western values necessitates advancement of the white race [27]. Accordingly, Jarrod Taylor (a prominent head of the AR) denounces multiculturalism, arguing that non-whites cannot maintain or contribute to Western civilization [25]. As recognised by Jardina, such ideas have contributed to the political divide on issues such as immigration and were able to be capitalised during the 2016 Trump candidacy [28]. Though many members claim their separatist views do not assume superiority of race [3], others stridently argue for the intellectual and cultural supremacy of whites [27]. As such, critics believe the AR to be a contemporary white supremacist movement [29].

### Proposed predictors of extreme political attitudes

In this section we outline a set of hypothesized predictors of PC and AR attitudes. Our choice of predictors was guided by the theoretical work on PC by Lukianoff & Haidt as well as the extensive empirical work on traditional political attitudes (i.e. moderate liberal and conservative attitudes) [10]. Specifically, Lukianoff and Haidt argue for the importance of overprotective parenting and social media use in the development of PC attitudes, whereas empirical studies on traditional political attitudes have confirmed the role of the Big Five personality traits (as outlined below) [10]. We have therefore chosen to focus on a broad set of predictors in this study; we believe this is appropriate given that our primary objective is to make an early contribution to understanding extreme political attitudes. We argue that, because so little is known about correlates of these attitudes, a useful starting point involves mapping extreme political attitudes onto a highly generalizable personality taxonomy (the Big Five) and drawing from the existing—albeit limited and largely untested—theoretical work in this area.

### News and social media

Over the previous 40 years, news reports have become increasingly negatively biased [30]. Consequently, individuals are tending to overestimate negative events and underestimate their personal safety [31]. Additionally, more people are retrieving news from social media sites: 68% of American adults occasionally getting news from social media, 20% saying that they do so often [32]. Social media sites allow users to curate their social network, which has seen news consumption become increasingly self-selected [33, 34]. While selectively engaging with certain outlets, users filter the newsfeed of others by ‘sharing’ stories to their social network. Politically consistent social media users are more likely to ‘follow’, ‘like’, and communicate with others that share their political attitudes, and to disconnect from users that express disparate opinions [35]. This has been argued to have created ‘echo chambers’, wherein information becomes increasingly uniform and limits one’s exposure to, and delegitimizes the moral claims of, disconfirming attitudes [36]. While popular thought supposes that intergroup contact diminishes contention, Bail et al. found that when partisans are exposed to antithetical views online, they become increasingly polarized [37]. This suggests online echo chambers decrease one’s leniency toward opposing views, increase confidence in pre-established viewpoints, and increase exposure to more extreme attitudes [38, 39].

Accordingly, mainstream outlets and psychologists have reported much online activity from the extreme left and right [40, 41]. While PC is widespread, several authors have observed AR proponents sanitize their ideology for public consumption through antagonistic memes satirizing PC culture and progressivism in general [13, 14]. This has been reported to have caused an influx of young subscribers who enter AR echo chambers and become introduced to racialist content. Therefore, we predict that extremists on the left and the right will spend more time on social media sites. Specifically, it is hypothesized that those reporting higher social media use will report higher levels of PCL (Hypothesis 1a), PCA (Hypothesis 1b), and WI attitudes (Hypothesis 1c).

### Overprotective parenting

Lukianoff and Haidt theorised the increase of PC can be attributed to generational changes in child rearing [10, 24]. While the typical childhood of Baby Boomers and Gen Xers was largely unsupervised, their Millennial and (especially) iGen children have been subject to a significant increase in parental surveillance (with a market for phone applications that allows constant monitoring of children, creating the so-called ‘electronic umbilical cord’) [42]. Parents are more likely to act on behalf of their children in ambivalent situations (with parents often speaking to college administrators for the concerns of their adult child) [43], and to disengage their child from any potentially dangerous activity (at least without adult supervision) [44, 45]. At school, pre-emptive safety measures have been realized with the enforcement of such things as zero-tolerance bullying policies and the insistence of formal, adult organized play times [46]. Further, children are provided an emotionally accommodating environment with awarding of ‘participation-trophies’. Consequently, overprotected children have less opportunity to independently explore the world: to experience, learn from, and realize they can survive failure.

Previous research indicates that overprotective parenting is associated with issues in anxiety [47], depression and lower life-satisfaction [48], worse academic performance, and more relational problems [49]. However, most problems associated with overprotective parenting can be largely explained through the child’s perception of their own competence and autonomy [48, 50]. In congruence with self-reports, overprotected children tend to be perceived by others as less resilient, capable, and independent [51]. As such, the ostensible increase in overprotective parenting practiced by Baby Boomers and Gen Xers may have made Millennial and iGen children more emotionally fragile, more likely to consider adverse content a threat, and more likely to seek an external authority to resolve their problems [52]. Thus, while ensuring the short-term welfare of children, overprotective parenting reduces a child’s development of resilience. Accordingly, without resilience to deal with problems, people consider adversity at any degree of severity as synonymous with violence, and therefore demand its immediate elimination. As such, it is hypothesised that perceptions of overprotective parenting will be negatively correlated with age (Hypothesis 2a), and resilience (Hypothesis 2b). Furthermore, it is hypothesised that perceptions of overprotective parenting (Hypothesis 3a) and low resilience (Hypothesis 3b) will predict PCA.

### Personality traits

We also consider personality traits as predictors of extreme political attitudes. Although the recent shift in political attitudes cannot be attributed to personality traits (i.e. overt levels of traits have not changed), it is likely that some individuals, based on their personality traits, will be more sympathetic to extreme attitudes. Indeed, academic literature demonstrates the association between personality traits and political preferences [53]. The current study therefore utilizes the most popular and empirically supported model of personality traits: the Big Five [54]. The Big Five is a descriptive model of personality that specifies the major, broad dimensions upon which people differ: Openness to Experience (Openness-Intellect), Extraversion, Conscientiousness, Agreeableness, and Neuroticism [55]. Although proven useful across a range of situations and contexts, researchers have extended the Big Five to account for higher and lower order factors with each of the five traits capturing the covariance of two lower-order traits (aspects) [56].

Specifically, Openness-Intellect describes one’s tendency to try new things and their engagement with ideas [56]. Openness-Intellect consists of aspect Openness, which details one’s interest in abstract content, and aspect Intellect, which measures one’s capacity for intellectual pursuits. Extraversion is the pleasure-seeking and positive affect dimension; as the behavioural mode of exploration, it manifests as social engagement. It includes aspects Assertiveness, which explains one’s sense of urgency and social dominance, and Enthusiasm, which measures sociability and outgoingness. Conscientiousness details one’s tendency to follow rules and pursue non-immediate goals. Its aspects are Industriousness and Orderliness, which detail one’s work ethic and desire for order, respectively. Agreeableness shows one's proclivity to be altruistic and cooperative with others. Comprising aspects Compassion and Politeness, it explains one’s sensitivity and interaction with others. Neuroticism is the measure of one’s sensitivity to threat and punishment. Ultimately, it is the negative affect dimension, and consists of aspects Volatility, measuring emotional reactivity, and Withdrawal, assessing susceptibility to negative emotion.

Political attitudes are highly explicative of two main traits: Openness-Intellect and Conscientiousness [17]. Liberals tend to be high in Openness-Intellect and low in Conscientiousness (conservatives showing the opposite disposition). However, analysis into personality aspects revealed that Agreeableness also contributes to political attitudes [53]. Specifically, liberals tend to be high in aspects Openness and Compassion, and low in Orderliness. Conservatives evince high levels of aspects Orderliness and Politeness, and low levels of Openness and Compassion.

Recently, Andary-Brophy found that PC attitudes are associated with trait Agreeableness, specifically aspect Compassion [11]. PCL is predicted by trait Openness-Intellect, specifically aspect Openness. PCA is predicted by high trait Conscientiousness, specifically aspect Orderliness. Therefore, compassionate ends largely motivate PC, however the means used to meet these ends, whether socially democratic or immediate and autocratic, are moderated either by Openness or Orderliness, respectively. It is predicted that PC adherents will evince more compassion to vulnerable groups and will differentiate as to whether they prioritize their consideration or protection of these groups. Specifically, it is hypothesized that high Compassion and Openness will predict PCL (Hypothesis 3a), and high Compassion and Orderliness will predict PCA (Hypothesis 3b).

Although no research has been conducted on the Big Five and WI attitudes, previous findings have shown that AR adherents display higher scores of right-wing authoritarianism (RWA) and social dominance orientation (SDO) [57]. As both RWA and SDO have been heavily studied, it may be possible to extend such findings to inform the hypotheses regarding WI attitudes. Such investigations have found a strong relationship between personality and authoritarian attitudes [58, 59]. Specifically, racially prejudiced attitudes are primarily predicted by low Openness-Intellect and Agreeableness, and high Conscientiousness [60]. Therefore, it is reasonable to conclude that people harboring prejudiced attitudes are less compassionate and have a need for conceptual and material organization. However, although manifesting as a rejection of minority groups, it remains possible that WI attitudes more appropriately represent an exaggerated acceptance (or protection) of white identity, and therefore, the aforementioned results apply to the AR only partially. Even so, these findings informed the hypotheses of the current study. It was hypothesized that WI attitudes would be predicted by low aspects Openness and Compassion, and high aspect Orderliness (Hypothesis 4).

### Moral absolutism

A final trait predictor of extreme political attitudes we consider is moral absolutism. Moral absolutism is the tendency to engage in rigid, ‘black-and-white’ moral thinking, in terms of others’ behavior [61]. Lukianoff and Haidt have argued that in the promotion of equality of outcome, advocates of PC necessarily apply a moral framework [10]. According to this moral framework, ‘good’ is defined as those promoting equal outcomes across groups, while ‘bad’ is defined as those in positions of power. Such a moral distinction allows advocates to make claims on the goodness of others character based on superficial characteristics. This superficial analysis then permits inference into others motives regardless of behavior. For example, guilt is not only attributable to those explicitly promoting inequality, but also to those deemed as non-committal benefactors of the system of inequality. Such a simple moral framework is likely to encourage proponents to adopt a morally dichotomous, ‘us versus them’, worldview.

This line of reasoning is directly applicable to WI attitudes. While PC defines morality on the basis of equity, WI appear to define it on group identity. Indeed, previous research has shown that morally dichotomous attitudes are associated with generalized prejudice [62]. Furthermore, those high in moral absolutism tend to regard others’ behaviours and attitudes as categorically ‘good’ or ‘bad’, resultantly providing moral license to directly oppose different viewpoints with force [63, 64].

As such, we suggest that moral absolutism is an antecedent to the adoption of extreme political attitudes. We argue that those with a tendency to make good-bad/right-wrong judgements about complex issues are more likely to uniformly accept one position and reject/oppose all others and consequently hold extreme views (rather than seeing both sides of an issue). As such, it is hypothesized that moral absolutism will significantly predict PCA (Hypothesis 5a) and WI attitudes (Hypothesis 5b).

We note that various other factors in this study potentially impact moral absolutism. For example, overprotective parenting and lower resilience in young people may serve to enhance dichotomous thinking in such individuals. We do not specifically test indirect effects in this study however, since our primary focus in this new research area is to identify predictors of extreme political attitudes. Nevertheless, we conduct hierarchical analyses to gain insight into the relative contribution of different predictors.

## Method

### Participants and design

Participants were a quota-based sample of 512 adults (Females = 264) between the ages of 18 and 84 (M = 45.7, SD = 16.9) living in the U.S. (see Table 1). This sample size was based on an a priori power analysis using G*Power 3.0 [65] specifying a regression model with small to medium effect size (f ^2^ = 0.08), 18 predictors (our largest regression included 13 predictors and 5 covariates), a power level of 0.9 and a p-value of 0.001. Output from G*Power specified that a minimum of 454 participants were required.

**Table 1:** Demographic characteristics of the participants.

Characteristic	Total Sample (%)
Gender	
Male	47.7
Female	51.6
Non-specific	0.8
Ethnicity	
African American	12.7
Asian	6.1
Caucasian	61.1
Hispanic	17.0
Native American	1.0
Other	2.1
Employment	
Yes	53.1
Full-time	38.9
Part-time	12.7
Casual	1.6
No	46.9
Highest level of education	
Year 10	12.7
Year 12	28.7
Certificate III/IV	16.4
Diploma/ Associate Diploma	8.8
Undergraduate Degree (Bachelor)	20.5
Postgraduate Degree (PhD, Masters, Graduate Diploma, etc)	12.9

The sample was recruited using the online panel research company, Qualtrics. Qualtrics was chosen over other research panels and MTURK because they offered the option of recruiting a more representative sample and high user control required to enhance data quality. To ensure that the sample was representative of the demographics in the US, quotas that reflected the demography of the national population were made on dimensions of age, ethnicity, gender, employment status, and education level. Qualtrics recruited participants according to these nationally representative quotas. Participants were informed of the nature of the study on an information sheet and provided informed consent by clicking on a button at the bottom of the page that continued them to the survey. The study utilized a cross-sectional, correlational design, whereby participants were asked to respond to an online self-report survey. For their participation, participants received ‘points’ from Qualtrics, which they can accrue and redeem for in-store rewards. Participants who completed the survey faster than one-third of the average completion time were removed from the sample. Ethics approval (approval number: 1800000544) was granted by the Queensland University of Technology (QUT) Human Research Ethics Committee (UHREC).

### Measures

Qualtrics software was used to conduct the online survey. Participants completed the survey via an anonymous link. The measures used in the survey are detailed here.

#### Politically correct attitudes

To assess PC, the 36-item PC scale (short version) [11] was used. The scale consists of two subscales: PCL-S and PCA-S (‘S’ stands for ‘short version’). The PCL-S scale is a 19-item scale that measures PCL, and the PCA-S scale has 17 items that measure PCA. Participants were asked to identify their level of agreement or their assessment toward the items, which were in the format of statements or definitions. For instance, items included statements such as “There are no biologically based differences in personality, talent, and ability to reason, between racial groups”. Because questions have different response options (Likert scale, and single choice items), a total PC score was calculated after averaging the standardized scores of the items (this procedure being repeated for the relevant items in each subscale). The current study found that the subscales PCL-S (α = .72) and PCA-S (α = .86) both reported acceptable Cronbach’s alphas.

#### White identarian attitudes

To measure WI attitudes, the WI Scale was developed. To create this scale, statements made by mainstream outlets regarding the AR as well as official statements made by recognized AR figureheads and endorsed by their followers were collected [3, 4, 25]. Such statements had three main themes: 1) a focus on ethnic identity, 2) a strong sense of white solidarity, and 3) a belief that whites are being displaced in the U.S. This method of scale development was consistent with that used by Andary-Brophy to develop the PC scale [11]. Overall, the questionnaire consists of 12 items, such as “Race is the foundation of identity” and “Whites are being forgotten and replaced by minorities in this country”. To complete the questionnaire, participants responded to statements on a 5-point Likert scale ranging from completely agree to completely disagree (the option of there being not enough background information was also provided and was considered synonymous with ‘neither agree nor disagree’ responses in statistical analyses). A total AR score was calculated after averaging the responses to each of the items. An exploratory factor analysis confirmed the presence of one primary factor and the internal reliability of the scale was satisfactory (α = .88).

#### Demographics and general information

Demographics were obtained with questions asking for age, gender, ethnicity, employment status, and highest level of education achieved. To allow for correlational analyses, demographic variables (gender, ethnicity, employment status, and education level) were coded as numbers prior to statistical analyses (i.e., gender: male = 1, female = 2; ethnicity: Caucasian = 1, other = 2; employment status: employed = 1, unemployed = 2; education: below grade 10 = 1, grade 12 = 2, trade/cert III/IV = 3, diploma/associate diploma = 4, bachelor degree = 5, post-graduate = 6). The amount of time spent on social media was assessed using a single-item measure. Participants were directly asked “On average, what is the approximate amount of time you spend on social media sites each day? Participants were given a set of 9 response options ranging from ‘none’ to “more than 6 hours”. Participants were told that social media sites include “Facebook, Twitter, YouTube, Snap chat, and other such sites.”

#### Overprotective parenting

To measure parental overprotection, the overprotection subscale of the Parental Bonding Instrument [66] was used. The overprotection subscale includes 13 items that measure self-reports of how controlling and invasive participants believed their parents to be up to the age of 16. Participants were required to respond to how such statements as my parent/s “felt I could not look after myself unless she/he was around” were representative of their childhood on a 4-point Likert scale, ranging from “very like” to “very unlike”. Scores were calculated using the averages of the items. The internal reliability satisfactory in the current study (α = .71).

#### Resilience

The Brief Resilience scale [67] was used to assess resilience. The scale consists of six items, which participants had to respond to on a 5-point Likert scale, ranging from strongly disagree to strongly agree. Questions included “I tend to bounce back quickly after hard times”. Resilience scores were calculated by averaging the responses to the items. Its authors have shown it to positively correlate with positive outcomes, and negatively with negative outcomes. In the current study, it had satisfactory internal reliability (α = .74).

#### Personality traits and aspects

The 100-item self-report Big Five Aspect Scale [57] was used to assess the Big Five and their respective aspects. The Big Five includes Openness-Intellect, Extraversion, Conscientiousness, Agreeableness, and Neuroticism. The aspects of Openness-Intellect include Openness to Experience and Intellect. The two aspects of Extraversion are Enthusiasm and Assertiveness. Conscientiousness includes aspects Industriousness and Orderliness. Agreeableness comprises aspects Compassion and Politeness. Lastly, the aspects of Neuroticism are Volatility and Withdrawal. The instrument consists of 10 scales (one for each aspect), each containing 10 items. Using a 5-point Likert scale ranging from strongly agree to strongly disagree, participants were asked to rate each item to the extent that they thought it was descriptive of them in general. After reversing the appropriate responses, scores for the 10 aspects were calculated by averaging the items. Scores of the five traits were calculated by averaging the scores of the two scales of their respective aspects. Previous research has validated it against standard Big Five scales, such as the Big Five Inventory (mean r = .88) and the Revised NEO Personality Inventory (mean r = .82) [56]. In this study, it had satisfactory internal reliability (mean α = .83).

#### Black-and-white moral thinking

Black-and-white moral thinking was assessed using the Moral Absolutism/Splitting items of the Attitude Toward Ambiguity construct [62]. The items consist of seven statements, such as “There is a right and a wrong way to do almost everything”. Participants expressed the degree to which they agree with the statements on a 7-point Likert scale ranging from strongly disagree to strongly agree. Moral absolutism scores were calculated after averaging the responses to the items. The scale was found to have high internal reliability in the current study (α = .91).

#### Traditional political attitudes

Left-right political attitudes were assessed using a single item. Participants were provided with the typical definition of liberal and conservative and were required to report their political views on a 5-point Likert scale ranging from very liberal to very conservative.

### Procedure

The survey was completed online via Qualtrics software. Once participants accessed their anonymous link, they were directed to a participant information page, which provided information relevant to the study. Upon reading the information sheet, tacit informed consent was provided in the participant continuing to the survey. Participation was voluntary and anonymous, and participants were able to withdraw from the study at any time (however this would forfeit their claim to the incentives from Qualtrics).

Upon commencing the survey, participants were asked to provide demographic information, and continued to the questionnaires. Participants completed the survey at their own pace, which generally took 24 minutes. Once they completed the survey, participants were provided the opportunity to make any comments about the survey and were thanked for their participation. The contact information of the research team was also provided if they wanted to make any further comments. The vast majority of participants who provided open-ended comments indicated that they found the survey enjoyable and thanked the research team for the opportunity to be involved.

### Data analysis

To investigate the hypotheses, several hierarchical multiple regression analyses were undertaken. Each regression was conducted using the same three-step procedure, with demographics in the first step, personality traits in the second, and parenting, resilience, moral absolutism, and social media use in the third step. This was done to control for demographics, and to assess whether the hypothesized non-Big Five predictors (parenting, resilience, moral absolutism, and social media) had incremental validity over personality traits. However, to include all participants in the analysis (i.e. employed and unemployed) employment status was not included in step 1. Also, to avoid issues of multicollinearity with the Big Five traits, personality aspects were not included the original analysis. Instead, subsidiary hierarchical multiple regression analyses were undertaken to assess the contribution of the personality aspects. Big Five traits were removed from the regression, and the personality aspects were introduced in the second step of the separate analyses (with the same hierarchical entrance of variables). To assess the unique contribution of the variables the reported values were attained from the third step of the model. The reported contributions of the non-personality predictors were taken from regression analyses with the Big Five traits unless specified otherwise.

## Results

### Political opinion in the U.S.

Analysis of the responses to the single item assessing traditional political attitudes (left vs right) showed that political opinion in the U.S. is normally distributed, with the largest portion of participants identifying as politically moderate (30.9%). To determine the proportion of participants holding ‘extreme’ attitudes, we looked at the frequencies of high scorers on PCL, PCA and WI scales. Because these variables were measured on a 1–5 response scale, we classified individuals obtaining a mean score of 4 or above as holding extreme attitudes. In other words, individuals were considered extreme scorers when they either agreed or strongly agreed (on average) to all items assessing each attitude. Using this criterion, we found that 8.2% of participants held extreme PCL attitudes, whereas 6.1% held extreme PCA attitudes. We also found that 14.1% of white participants held attitudes typical of the AR (see Table 2).

**Table 2:** Overall level of agreement with extreme political attitudes.

	Strongly Disagree	Disagree	Neutral	Agree	Strongly Agree
PCL	0.8	6.2	61.2	30.0	1.8
PCA	3.3	34.8	43.9	15.9	2.1
WI	3.1	12.3	28.7	13.4	3.5

Numbers represent the participant’s average response to questions in each scale. They do not represent adherence to these attitudes. WI reports prevalence among white participants as a percentage of the total sample.

To provide a clearer picture of the extent of extreme political attitudes in the U.S., we next provide an overview of frequencies for a selection of questions from each scale. Regarding PCL attitudes, 2 in 10 participants agreed or strongly agreed that retail stores should avoid using the word ‘Christmas’ in advertising campaigns; 7 in 10 participants believed that newspapers should have some degree of screening for offensive, racist or sexist language/ideas. Regarding PCA attitudes, approximately 8 in 10 participants believe a professor should be punished in some form for using a racist, sexist or homophobic slur when teaching a class; 3 in 10 participants agreed or strongly agreed that an alleged perpetrator of sexual assault should have to prove their innocence.

Regarding WI attitudes, approximately 2 in 10 white participants agreed or strongly agreed that racially or ethnically defined states are legitimate and necessary; 3 in 10 white participants agreed or strongly agreed that there was a progressive conspiracy against white identity; 3 in 10 white participants agreed or strongly agreed that whites are being forgotten or replaced by minorities in this country.

### Bivariate correlations among focal variables

Bivariate correlational analysis was run to investigate relationships amongst demographics and extreme political attitudes (see Table 3), and hypothesised predictors. Hypotheses were tested based on standard errors using a large number of bootstrapped samples (1000 samples). The analysis revealed that age is significantly negatively correlated with parental overprotection, *r* = -.177, *p* < .001. Parental overprotection was also negatively correlated with resilience, *r* = -.218, *p* < .001. Further, age was significantly correlated negatively with social media use, *r* = -.313, *p* < .001, and positively with resilience, *r* = .218, *p* < .001. PCL was shown to be positively correlated with PCA, *r* = .428, *p* < .001, and non-significantly correlated with WI, *r* = .001, *p* < .996. However, PCA and WI attitudes were positively correlated, *r* = .48, *p* < .001.

**Table 3:** Means, standard deviations, bivariate correlations of PCL, PCA, WI, and demographic variables.

	M	SD	1	2	3	4	5
1. PCL	2.35	.56	(.72)				
2. PCA	1.73	.84	.428**	(.86)			
3. WI	3.1	.85	.001	.48**	(.88)		
4. Age	45.6	17.8	.023	-.305**	-.199**		
5. Gender			-.011	-.053	-.226**	-.051	
6. Education Level			.248**	.102*	.003	.101*	-.166**

*p < .05

**p < .001; All p values are two-tailed. Scale reliability are Cronbach’s alpha’s, shown in parentheses. All correlations with WI included white participants only. Significance tests were based on 1000 bootstrapped samples.

### Predicting PCL

A hierarchical regression analysis was used to assess predictive relationships between hypothesized IV’s and PCL. Demographic variables were entered in the first step, personality aspects were added to the second step, and non-Big Five variables (social media use, overprotective parenting, resilience, and moral absolutism) were entered to the third step. At the first step, demographics significantly predicted PCL, *R*^*2*^ = .063, *F*(5, 506) = 6.8, *p* < .001. At the second step, personality aspects collectively predicted PCL, *ΔR^2^* = .088, *F*(10, 498) = 5.14, *p* < .001 (step 2). At the third step, predictors did not significantly contribute to the variance in PCL, *ΔR^2^* = .036, *F*(4, 492) = .5.39, *p* < .001. Overall, the model explained 18.7% (15.5% adjusted) of the variance in PCL.

The unique contribution of the variables was assessed at the third step of the regression model. Unique effects were observed for compassion, β = .26, *p* < .001, Intellect, β = .19, *p* = .002, moral absolutism, β = .13, *p* = .005, and social media use, β = .14, *p* = .002 (see Table 4).

**Table 4:** Step 3 of the hierarchical multiple regression of BFAS, parenting, and personal factors with PCL.

		*B*	*SE*	95% CI	β	*R*^*2*^	Δ *R*^*2*^	*sr*^*2*^
BFAS						.151***	.088***	
	Volatility	-.01	.04	[-.08, .06]	-.01			.00
	Withdrawal	.04	.05	[-.05, .13]	.07			.00
	Compassion	.17	.04	[.08, .25]	.26***			.02
	Politeness	-.04	.04	[-.13, .04]	-.07			.00
	Industriousness	-.07	.04	[-.15, .02]	-.12			.00
	Orderliness	.00	.04	[-.08, .08]	.00			.00
	Enthusiasm	-.03	.04	[-.12, .05]	-.04			.00
	Assertiveness	-.03	.04	[-.11, .05]	-.04			.00
	Intellect	.13	.04	[.05, .21]	.19**			.02
	Openness	.03	.04	[-.06, .11]	.03			.00
Other						.187***	.036	
	Overprotectiveness	.00	.03	[-.06, .07]	.00			.00
	Resilience	.03	.03	[-.03, .09]	.05			.00
	Moral Absolutism	.04	.01	[.01, .06]	.13**			.01
	Social Media	.02	.01	[.01, .04]	.14**			.01

* p < .05

** p < .007

*** p < .001; All p values are two-tailed.

### Predicting PCA

A hierarchical regression model was also used to investigate hypothesized predictors of PCA. Demographics were added to the first step, personality aspects to the second, and the non-Big Five variables to the third. At the first step, demographics significantly predicted PCA, *R*^*2*^ = .121, *F*(5, 506) = 13.96, *p* < .001. At the second step, personality aspects provided significant explanatory power, *ΔR^2^* = .068, *F*(10, 496) = 4.18, *p* < .001 (step 2). At the third step, predictors collectively accounted for variance in PCA, *ΔR^2^* = .171, *F*(4, 492) = 32.82, *p* < .001. Overall, the model explained 36.0% (33.6% adjusted) of the variance.

Again, individual contribution was assessed at the third level. Aspects of Agreeableness were observed as significant predictors in opposite directions; PCA was positively predicted by Compassion, β = .21, *p* = .001, and negatively by Politeness, β = -.24, *p* < .001. Overprotection, β = .16, *p* < .001, and resilience, β = -.10, *p* = .041, reported significant effects. Further, moral absolutism, β = .37, *p* < .001, and social media, β = .10, *p* = .012, were significant predictors (see Table 5).

**Table 5:** Step 3 of the hierarchical multiple regression of BFAS, parenting, and personal factors with PCA.

		*B*	*SE*	95% CI	β	*R*^*2*^	Δ *R*^*2*^	*sr*^*2*^
BFAS						.151***	.088***	
	Volatility	.03	.04	[-.05, .11]	.04			.00
	Withdrawal	-.07	.06	[-.17, .04]	-.09			.00
	Compassion	.18	.05	[.07, .29]	.21***			.01
	Politeness	-.20	.05	[-.30, -.10]	-.24***			.02
	Industriousness	-.01	.05	[-.11, .10]	-.01			.00
	Orderliness	.01	.05	[-.08, .11]	.01			.00
	Enthusiasm	.03	.05	[-.07, .13]	.03			.00
	Assertiveness	.07	.05	[-.03, .16]	.07			.00
	Intellect	-.04	.05	[-.14, .06]	-.04			.02
	Openness	-.05	.05	[-.15, .05]	-.05			.00
Other						.187***	.036	
	Overprotectiveness	.17	.04	[-.09, .25]	.16***			.02
	Resilience	-.08	.04	[-.16, -.00]	-.10*			.00
	Moral Absolutism	.15	.02	[.12, .18]	.37**			.11
	Social Media	.02	.01	[.01, .04]	.10**			.01

* p < .05

** p < .007

*** p < .001; All p values are two-tailed.

### Predicting AR

A 3-step hierarchical regression analysis was also undertaken to investigate predictors of WI attitudes. Demographics were put in step one, personality aspects in step two, and non-Big Five variables in step three. At the first step, demographic variables significantly predicted WI attitudes, *R*^*2*^ = .094, *F*(4, 308) = 7.95, *p* < .001. At the second step, the aspects collectively explained a significant amount of variance, *ΔR^2^* = .176, *F*(10, 298) = 7.19, *p* < .001. At the third step, variables significantly predicted WI attitudes, *ΔR^2^* = .319, *F*(4, 294) = 57.13, *p* < .001. The total model explained 58.9% (56.4% adjusted) of the variance.

Analysis into the unique contribution revealed that the effects of Politeness, β = -.22, *p* = .001, and Openness, β = -.10, p = .038, were negative, while the effect of Orderliness, β = .10, *p* = .047, was positive. Further, moral absolutism, β = .61, *p* < .001, and overprotective parenting, β = .09, *p* = .018, were positive predictors (see Table 6).

**Table 6:** Step 3 of the hierarchical multiple regression of BFAS, parenting, and personal factors of WI attitudes.

		*B*	*SE*	95% CI	β	*R*^*2*^	Δ *R*^*2*^	*sr*^*2*^
BFAS						.27***	.176***	
	Volatility	.03	.07	[-.10, .16]	.03			.00
	Withdrawal	.00	.09	[-.18, .17]	.00			.00
	Compassion	.00	.09	[-.17, .16]	.00			.00
	Politeness	-.28	.08	[-.44, -.12]	-.22**			.02
	Industriousness	-.04	.09	[-.21, .13]	-.03			.00
	Orderliness	.16	.08	[.00, .32]	.10*			.00
	Enthusiasm	.09	.08	[-.07. .25]	.06			.00
	Assertiveness	.08	.08	[-.08, .23]	.05			.00
	Intellect	-.08	.08	[-.24, .08]	-.06			.00
	Openness	-.16	.08	[-.31, -.01]	-.10*			.01
Other						.589***	.319***	
	Overprotectiveness	.14	.06	[.02, .26]	.09*			.01
	Resilience	.03	.06	[-.09, .16]	.03			.00
	Moral Absolutism	.36	.03	[.31, .41]	.61***			.28
	Social Media	.02	.02	[-.01, .05]	.05			.00

* p < .05

** p < .003

*** p < .001; All p values are two-tailed.

## Discussion

Previous research claims increased political polarization in the U.S., with an ostensible mass attitudinal migration toward PC on the left, and the AR on the right [2]. Although highly covered in mainstream media and theorized by prominent social psychologists, academic literature on these attitudes is scarce. The current study represents the first in-depth investigation into the psychological correlates of PC and the AR. Using a large quota-based U.S. sample, we found extreme attitudes represent a significant minority of attitudes in America. Accordingly, most participants were indifferent, disagreed, or strongly disagreed with the extreme left and right. This study confirmed previous research [11] that PC is a multidimensional construct, and also provided evidence that adherents of the extreme left and right share certain traits.

We observed the effect of social media on political attitudes was different for extreme left and extreme right attitudes. While PCL and PCA were significantly predicted by social media use, WI attitudes were not. As most participants (56.4%) reported that Facebook was their most used site, such findings are in accordance with previous research reporting a disproportionate amount of politically left content on Facebook [68]. Liberals are also more likely to use social media in general [69]. With more liberal users and content, it is likely that Facebook has more liberal echo chambers, and therefore increases the probability of engaging with leftist reverberations.

Secondly, as the first investigation into the hypotheses of Lukianoff and Haidt, this study provides preliminary evidence that changes in parenting have contributed to extreme left attitudes [10]. Generational changes in overprotective parenting were observed, with younger people more likely to report having overprotective parents. Further, overprotective parenting and low levels of resilience differentiated whether extreme attitudes on the left manifested as PCL or PCA (contributing to PCA but not PCL).

Thirdly, aligning with previous research, personality aspects significantly predicted political attitudes. PC partially manifested from trait Agreeableness, with aspect Compassion predicting PCL and PCA, and low aspect Politeness predicting PCA. However, against previous findings [11], aspects Openness and Orderliness were non-significant predictors of PCL and PCA, respectively. The discrepant findings of aspect Openness may be explained by the different regression analyses undertaken in this study, as compared to Andary-Brophy. After performing a correlational analysis, Andary-Brophy excluded variables that reported non-significant correlations with PC from later regression analyses to avoid issues of statistical singularity [11]. As preliminary investigation showed this was not problematic in the current study, all personality aspects were included in regression models. Further, the current study reported a significant bivariate correlation between Openness and PCL. Therefore, while Andary-Brophy reported significance after controlling for some other aspects, after controlling for all variables in the current study, the unique contribution of Openness was non-significant. However, the bivariate correlation between Orderliness and PCA was non-significant, which confuses the discrepancy.

Fourthly, WI attitudes were negatively predicted by trait Openness-Intellect, specifically aspect Openness; and trait Agreeableness, specifically aspect Politeness. However, the contribution of trait Conscientiousness (despite positive contribution from aspect Orderliness) was non-significant (see Table 7). As previous research has shown RWA attitudes are largely explained by Conscientiousness [60], the results suggest WI attitudes are not explicative of typical far right-wing attitudes. Indeed, at the trait level the AR displayed the opposite personality as PCL, with PCL positively predicted by traits Openness-Intellect and Agreeableness. As recognised by Andary-Brophy, PCL is characterised by the desire for the inclusion of all ethnic groups, and therefore truly represents the liberal compassion for recognised, disadvantaged groups [11]. Against this, the AR is marked by racial exclusivity and a belief in societal prosecution against whites. It is possible that progressive advocacy of ethnic diversity serves as confirmation for AR adherents of the narrative that whites are being displaced. This narrative then motivates their defence of white identity through anti-leftism. This suggests that the AR may be more appropriately conceptualised as the extreme opposition to progressivism.

**Table 7:** Step 3 of hierarchical multiple regression of big-5 traits, parenting, and personal factors.

Attitude	Step		*B*	*SE*	95% CI	β	*R*^*2*^	Δ *R*^*2*^	*sr*^*2*^
PCL	2	Neuroticism	.05	.04	[-.02, .12]	.09	.13***	.06***	.00
		Agreeableness	.11	.04	[.03, .19]	.16**			.01
		Conscientiousness	-.06	.04	[-.15, .03]	-.08			.00
		Extraversion	-.04	.05	[-.13, .06]	-.04			.00
		Openness-Intellect	.18	.05	[.09, .27]	.21***			.02
	3	Overprotectiveness	.01	.03	[-.06, .08]	.01	.16***	.04***	.00
		Resilience	.02	.03	[-.04, .09]	.04			.00
		Moral Absolutism	.03	.01	[.01, .06]	.11**			.01
		Social Media	.02	.01	[.01, .04]	.16***			.02
PCA	2	Neuroticism	.03	.05	[-.06, .12]	.04	.16**	.04***	.00
		Agreeableness	-.06	.05	[-.16, .04]	-.06			.00
		Conscientiousness	.00	.05	[-.10, .11]	.00			.00
		Extraversion	.18	.06	[.06, .30]	.16**			.01
		Openness-Intellect	-.05	.06	[-.17, .06]	-.04			.00
	3	Overprotectiveness	.18	.04	[.10, .26]	.17***	.34***	.18***	.03
		Resilience	-.07	.04	[-.15, .00]	-.09			.00
		Moral Absolutism	.14	.02	[.11, .17]	.35***			.10
		Social Media	.03	.01	[.01, .05]	.13**			.01
WI	2	Neuroticism	.09	.07	[-.05, .23]	.08	.24***	.22***	.00
		Agreeableness	-.30	.08	[-.46, -.14]	-.21***			.02
		Conscientiousness	.13	.08	[-.03, .29]	.09			.00
		Extraversion	.25	.09	[.06, .43]	.15**			.01
		Openness-Intellect	-.22	.09	[-.40, -.04]	-.12*			.01
	3	Overprotectiveness	.15	.06	[-.03, .27]	.10*	.58***	.56***	.01
		Resilience	-.01	.06	[-.10, .14]	-.02			.00
		Moral Absolutism	.36	.03	[.31, .40]	.61***			.29
		Social Media	.03	.02	[-.00, .06]	.07			.00

* p < .05

** p < .01

*** p < .001; All p values are two-tailed.

### Practical implications

A major outcome of extreme political views is violence. Examples include the aforementioned Charlottesville rally and violent protests against ‘controversial’ speakers on college campuses. From a practical standpoint, our findings help us understand the underlying causes of specific instances of violent behaviours and may assist in reducing relevant instances of violent behaviour. Our generally strong values for adj. R square suggest the set of variables in this study are highly relevant for extreme political attitudes.

As seen in Table 3, PCA and WI attitudes are positively correlated. This suggests that a common trait may explain their engagement in violent protests. Indeed, it is reasonable to suspect the violent protest behaviour typical of these groups may be partially explained by the common trait of moral absolutism. Black-and-white moral thinking has been shown to be associated with a willingness to support violence [70]. Black-and-white moral thinking castigates opponents as morally decrepit and supports engagement in extreme intervention against antithetical views [64]. As moral absolutism is characterised by an unquestioning assumption of one’s already established moral claims [71], this suggests that exposure to the legitimate moral claims of opposing views may reduce one’s certainty. Without absolute conviction in one’s preconceptions, adherents of these groups may be less likely to engage in extreme behaviours in defence of them.

### Limitations

A limitation in the present study is the cross-sectional design. While permitting investigation into correlations, this study cannot inform causal mechanisms. Second, this study used retrospective self-reports to assess parental overprotection. As recognised by Schwarz [72], in responding to retrospective self-reports of behaviours that occurred frequently (as in the Parenting Bonding Instrument), participants do not have an accurate picture but rely heavily on estimation strategies to recount such information [69]. As such, responses may be unreliable. Third, while the total sample size is sufficient to generalise findings to the population and is consistent with previous polling data on U.S. agreement with these attitudes [9], we caution the interpretation of the demographic data. Because this is the first look into the demographics of these attitudes, we maintain this data is interesting, however it is possible that the sample sizes of each group may not be sufficient to generalise to the population.

Finally, social media use was measured using a single self-report item. However, the use of a single item scales can be justified given the large scope of the current research [73]. Further, as recognised by Fuchs and Diamantopoulus, when assessing explicit and generally understood constructs, single item measures can provide valid and reliable responses. Because ‘social media use’ was specifically defined at the start of the survey and can be considered general knowledge, a single-item assessment is appropriate [74].

## Future research

It is important that future studies address the identified limitations. It is recommended that future research assess the psychometric properties of the WI scale, and its validity in properly tapping into AR attitudes. As explained in the methods section, the items were developed after collating commonly used phrased by AR adherents. Even so, some of the questions are also typical of traditional conservative ideas (for instance, “Marriage should only be allowed between a man and a woman”). This suggests that the scale may have incompletely approximated WI attitudes, advertising the need to properly define the construct. It is possible those in agreement with standard conservative attitudes are more likely to engage with people espousing more extreme attitudes of the political right and therefore display a more sympathetic interpretation of their plights.

Second, while this study provides insight into the prevalence of PC and WI attitudes in the U.S., future studies should assess the prevalence of such attitudes in other countries. This will not only report the spread of these attitudes outside of the U.S. but may also elucidate how different cultural environments contribute to the development of such attitudes.

Third, given the scope of this study, more complicated pathways were unable to be investigated. For instance, the contribution social media has on adherence to extreme political attitudes may be moderated by the particular social media site. Investigation into potential moderating effects from different social media platforms is recommended.

Further research should also continue the investigation into the hypotheses of Lukianoff and Haidt [10]. Specifically, research should investigate how overprotective parenting contributes to PCA via other developmental pathways. As previously mentioned, the results of the present study suggest that overprotective parenting (while not necessarily lowering resilience) reinforces the assumption that requesting intervention from authoritative parties will solve personal adversities. It is therefore recommended that future research investigate how variables such as psychological entitlement contribute to PCA.

## Conclusion

The current study is the first in-depth investigation into the prevalence and developmental associates of PC and the AR. The study found that although PC and WI attitudes are prevalent, the majority of Americans disagree with extreme political attitudes on both the political left and the right. Further, generational changes in social media use and overprotective parenting contributed to the development of PC. However, social media did not contribute to WI attitudes. Personality trait Agreeableness was shown to significantly explain extreme political attitudes, with high Compassion separating PC from WI attitudes, and low Politeness separating PCA from PCL. At the trait level, Agreeableness and Openness-Intellect revealed PCL and AR to be antonymous. Lastly, authoritarian attitudes in the political left and right were predicted by moral absolutism.

These findings provide clear evidence that PC and AR are valid terms representing the far ends of political thought in the U.S. Further, this study contributes to the literature, finding that political differences are not insubstantial, but are largely explained by one’s environment, upbringing, and individual disposition.

## Supporting information

S1 File (DOCX)

S1 Data (SAV)
