# Supplementary figures and images for: Political correctness and the alt-right: The development of extreme political attitudes
Source: PLoS One. 2020 Oct 7;15(10):e0239259. doi: 10.1371/journal.pone.0239259 (PMC7540844; doi:10.1371/journal.pone.0239259)

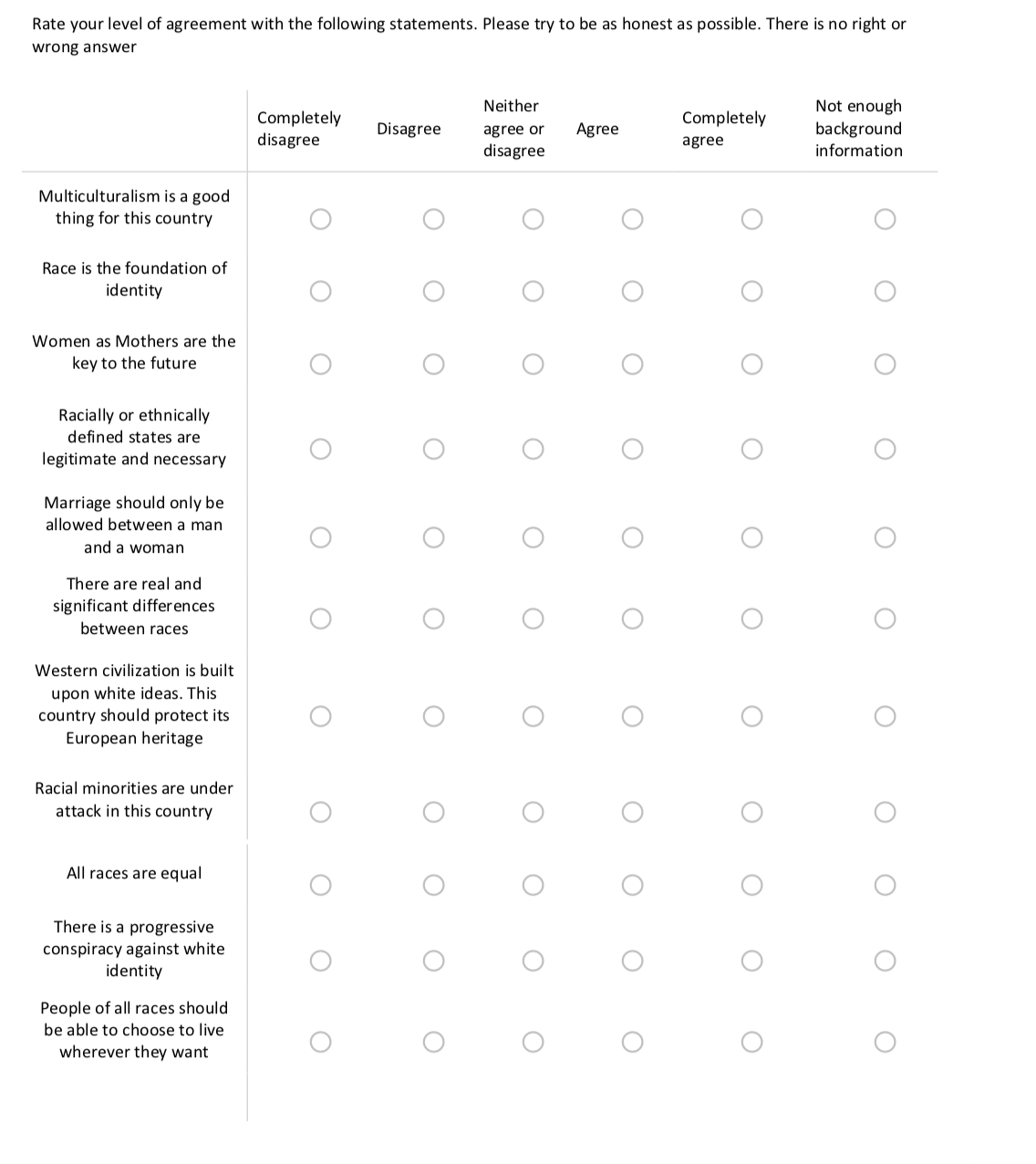

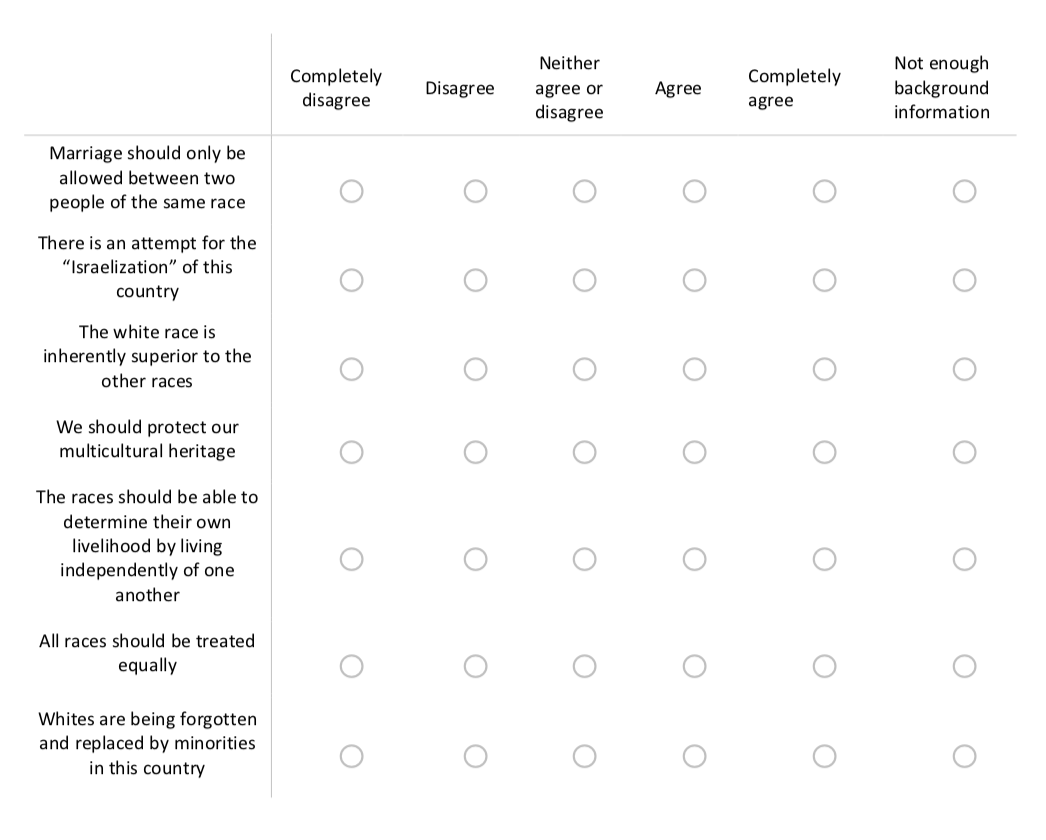

Supplement: S1 File — (DOCX) [file pone.0239259.s001.docx]
